# The influence of aortic stiffness on carotid stiffness: computational simulations using a human aorta carotid model

**DOI:** 10.1098/rsos.230264

**Published:** 2024-03-20

**Authors:** Marjana Petrova, Yujie Li, Alireza Gholipour, Hosen Kiat, Craig S. McLachlan

**Affiliations:** ^1^ Centre for Healthy Futures, Torrens University Australia Surrey Hills, New South Wales 2010, Australia; ^2^ Faculty of Medicine and Health, University of Sydney, Sydney, New South Wales 2006, Australia; ^3^ Faculty of Medicine, Health and Human Sciences, Macquarie University, Sydney, New South Wales 2109, Australia; ^4^ School of Rural Medicine, University of New South Wales, New South Wales 2640, Australia

**Keywords:** aortic stiffness, carotid stiffness, computer simulation models

## Abstract

Increased aortic and carotid stiffness are independent predictors of adverse cardiovascular events. Arterial stiffness is not uniform across the arterial tree and its accurate assessment is challenging. The complex interactions and influence of aortic stiffness on carotid stiffness have not been investigated. The aim of this study was to evaluate the effect of aortic stiffness on carotid stiffness under physiological pressure conditions. A realistic patient-specific geometry was used based on magnetic resonance images obtained from the OsiriX library. The luminal aortic–carotid model was reconstructed from magnetic resonance images using 3D Slicer. A series of aortic stiffness simulations were performed at different regional aortic areas (levels). By applying variable Young's modulus to the aortic wall under two pulse pressure conditions, one could examine the deformation, compliance and von Mises stress between the aorta and carotid arteries. An increase of Young's modulus in an aortic area resulted in a notable difference in the mechanical properties of the aortic tree. Regional deformation, compliance and von Mises stress changes across the aorta and carotid arteries were noted with an increase of the aortic Young's modulus. Our results indicate that increased carotid stiffness may be associated with increased aortic stiffness. Large-scale clinical validation is warranted to examine the influence of aortic stiffness on carotid stiffness.

## 1. Introduction

Carotid stiffness has been associated with cerebrovascular disease, cognitive impairment, and more recently incident depressive symptoms [1–3]. Disease risk with the ageing process is associated with generalized stiffening of the larger arterial vessels and the degree of arterial stiffness has been shown to be a predictor of future cardiovascular events and all-cause mortality, independent of traditional risk factors [1].

The standard interpretation of the relationship between vascular stiffness and blood pressure is that it increases blood pressure (BP). Pulse pressure (PP) increases pulsatile aortic wall stress, advancing elastic fibre degeneration [4,5]. Importantly, several studies have demonstrated that increased local carotid and aortic stiffness levels in normotensive individuals are associated with an increased risk of incidental higher BP and progressive development of hypertensive BP over time [4,6,7]. Additionally higher carotid–femoral pulse velocity in adolescents has been associated with obesity and hypertension later in life, suggesting a bidirectional relationship between hypertension and arterial stiffness [8,9].

The stiffness of the arterial wall is nonuniform along the arterial tree. Oxygenated blood is propagated through the elastic aorta toward stiffer muscular peripheral arteries, creating an impedance gradient ascending progressively from the heart to the peripheral arteries [10]. In the healthy and younger arterial system, the ascending impedance creates a wave reflection, reducing distal energy entering the microcirculation [10,11]. With large vessel stiffening, the microvascular structures may be sensitive to the PP and mean arterial pressure (MAP), resulting in either pathological end organ cardiac hypertrophy or increased peripheral vascular resistance if MAP is elevated beyond normal healthy physiology [10].

In diabetic and hypertensive patients, the aorta stiffens significantly more with age than local carotid stiffness [12,13]. In recent years new methods and techniques have been developed which allow the examination of aortic and local stiffness. However, these methods provide a limited evaluation of the arterial stiffness in each segment of the arterial tree. For example, pulse wave velocity (PWV), a gold standard for the measurement of central aortic stiffness, provides only an estimate of aortic stiffness as PWV represents a sum of the biomechanical and mechanical properties of different vascular walls located between the two measurement points of reference [14]. The relationship between aortic and carotid stiffness also remains undetermined despite emerging interest for their role in pathogenetic mechanisms of cardiovascular diseases. Computer simulations changing aortic stress in real time for a case study have not been undertaken to understand biomechanical changes in the carotid artery wall. This study aims to address these gaps to evaluate the relationship between aortic and local carotid stiffness using software simulation models derived from actual patient imaging and the ability to analyse this complex behaviour under physiological conditions.

## 2. Material and methods

### 2.1. Modelling of aorta and carotid artery

**Figure 1.**
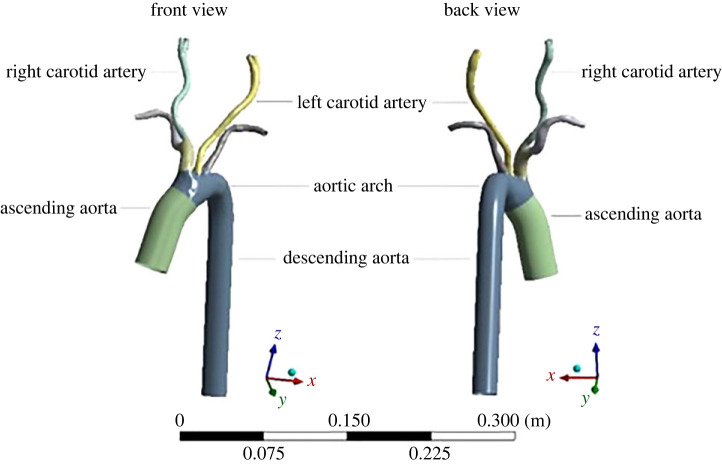
Three-dimensional simulation geometry of the patient-specific aorta and carotid arteries.

This study examined a realistic patient-specific vascular geometry of the aorta and carotid arteries, adapted from a case in the OsiriX library (https://www.osirix-viewer.com/resources/dicom-image-library/) where magnetic resonance imaging (MRI) was available. Using 3D Slicer, realistic geometry was reconstructed in the systolic phase of the cardiac cycle [15]. The lumen boundary of the vascular regions of interest was then extracted and reconstructed into a virtual vascular geometry, which included a patient-specific aorta and carotid arteries. The outcome was a vascular system for use in the following computational simulations (figure 1). From published clinical studies, we defined arterial wall thicknesses for aorta, brachiocephalic artery, carotid arteries, and subclavian arteries as 2.6, 1.5, 1.0 and 1.0 mm, respectively [16–18].

### 2.2. Simulation settings

The geometry was discretized with three-dimensional hexahedral elements to create a computational mesh with 36 158 elements in total [19]. This mesh condition was then re-examined and appeared to be responsive to variable simulation parameters.

Firstly, a two-step series of simulations were performed across different Young's modulus set points for the entire aorta and carotid. This was to investigate the effect of aortic stiffness on carotid arteries, as shown in tables 1 and 2. To achieve this outcome, first, a realistic pressure wave was applied to the inner wall for the entire geometry, with fixed supports defined at all openings of the model. A value of 40 mmHg indicates a pulse pressure in a healthy individual [21] and as little as 10 mmHg increase can increase the cardiovascular risk as much as 20% [20]. Two diverse levels of the pressure wave (pulse pressure) were examined for each step of the simulation: PP1—indicating a normal pulse pressure of 5994 Pa (42 mmHg); and PP2—indicating an increased PP of 7639 Pa (57 mmHg). Linear transient simulations of a single-layered aorta–carotid model were then performed in ANSYS Workbench 2020 R2 (ANSYS, USA).

**Table 1.** Assumed stiffness parameters for aorta and carotid arteries adapted from Gao *et al.* [20].

	aorta		carotid	
	Young's modulus, *E* (MPa)	Poison's ratio	Young's modulus, *E* (MPa)	Poison's ratio
Case 1	0.80	0.45	1.00	0.45
Case 2	1.00	0.45	1.00	0.45
Case 3	1.20	0.45	1.00	0.45
Case 4	1.40	0.45	1.00	0.45
Case 5	1.60	0.45	1.00	0.45

**Table 2.** Stiffness parameters for aorta and carotid arteries.

	aorta				carotid	
	ascending	arch	descending	Poison's ratio		
	Young's modulus, *E* (MPa)				Young's modulus, *E* (MPa)	Poison's ratio
Case 1	0.80	0.90	1.00	0.45	1.00	0.45
Case 2	1.00	1.10	1.20	0.45	1.00	0.45
Case 3	1.20	1.30	1.20	0.45	1.00	0.45
Case 4	1.40	1.50	1.40	0.45	1.00	0.45
Case 5	1.60	1.70	1.80	0.45	1.00	0.45

### 2.3. Structural analysis parameters

A variety of mechanical properties were analysed to examine the effect of aortic stiffness on carotid arteries.

Deformation—The deformation of the arterial wall was calculated with the following formula: *U* = √*Ux*2 + √*Uy*2 + √*Uz*2, where *U* = total deformation and *Ux*, *Uy* and *Uz* are the three component deformations.

Compliance—The classic definition of arterial compliance is the blood volume change relative to the distending pressure. However, the direct measurement of aortic compliance is challenging as there are no simple clinical methods to estimate the local changes in the blood volume.

To consider the compliance of the whole aortic system, the volume change (Δ*V*) of the systolic–diastolic status for the entire geometry and each separate section are respectively calculated over the pulse pressure (Δ*P*): compliance = Δ*V*/Δ*P* [22].

Von Mises stress—The von Mises stress is used to predict the yielding of materials under complex loading from the results of uniaxial tensile tests: *J*2 = *K*^2^, where *K* is the yield stress of the material in pure shear.

## 3. Results

Clinically, it is common to refer to a patient's ‘stiff arteries’. Simplified stiffness is a general term describing the vessel's resistance to deformation. However, defining the stiffness of arterial blood vessels can also be challenging as no single number or index can describe the complex mechanical behaviour of the vessel.

### 3.1. Variable stiffness for the entire aorta on a fixed compliance of the carotid

Firstly, we developed a series of aorta and carotid artery simulations to examine the increasing effect of aortic stiffness on normal carotid arteries. Five different values of Young's modulus for the whole aorta were applied (0.8–1.8 × 10^6^ Pa), while the Young's modulus for both carotid arteries remained constant (1.0 × 10^6^ Pa).

We examined the aortic and carotid deformation, compliance, and von Mises stress under standard PP = 5994 Pa, followed by a variation of the PP = 7639 Pa simulating normal and increased PP in a physical environment.

**Figure 2.**
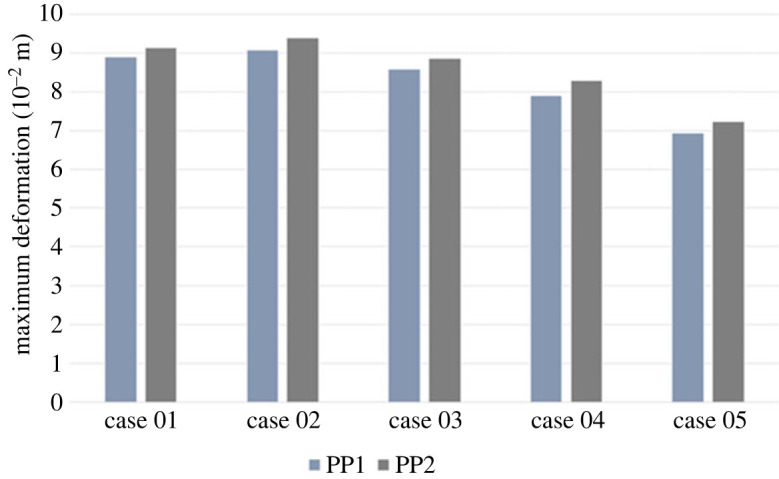
Deformation of entire aorta and carotid arteries with increasing Young's modulus under two pulse pressure conditions: PP1 = 5594 Pa; PP2 = 7369 Pa.

Figure 2 illustrates the deformation of the aorta and carotid artery, with a maximum and minimum value of Eaorta modulus set for our simulation under the two different PPs applied.

With the increase of Young's modulus, our model's deformation of the aorta and carotid arteries experienced a decrease during the entire cardiac cycle. Specifically, we demonstrate that less stiff aorta (Eaorta = 0.8 × 10^6^ Pa) experiences a maximal deformation of 9.14 × 10^−2^ (m) compared to the stiffer aorta (Eaorta = 1.6 × 10^6^ Pa), with maximum deformation of 7.23 × 10^−3^ (m).

We further investigated the deformation of the aorta and carotid arteries under increased PP. Similarly, as Young's modulus increases, the deformation of the aorta and carotid arteries in our simulation series decreases during the cardiac cycle. In particular, the aortic simulation with the lower value of Eaorta = 0.8 × 10^6^ Pa demonstrated maximum deformation of 8.91 × 10^−2^ (m) and a value of Eaorta = 1.6 × 10^6^ Pa demonstrated maximum deformation of 7.23 × 10^−3^ (m).

The distribution of maximum deformation across the aortic tree is presented in the electronic supplementary material (figure S1).

### 3.2. Compliance

Graphic illustration in the compliance of different sections in the model affected by Young's modulus under two pulse pressure conditions for the aorta is presented in the electronic supplementary material (figure S2). Across 5 cases under both pressure conditions, aortic and carotid compliance decreases with the increase of the Young's modulus.

**Figure 3.**
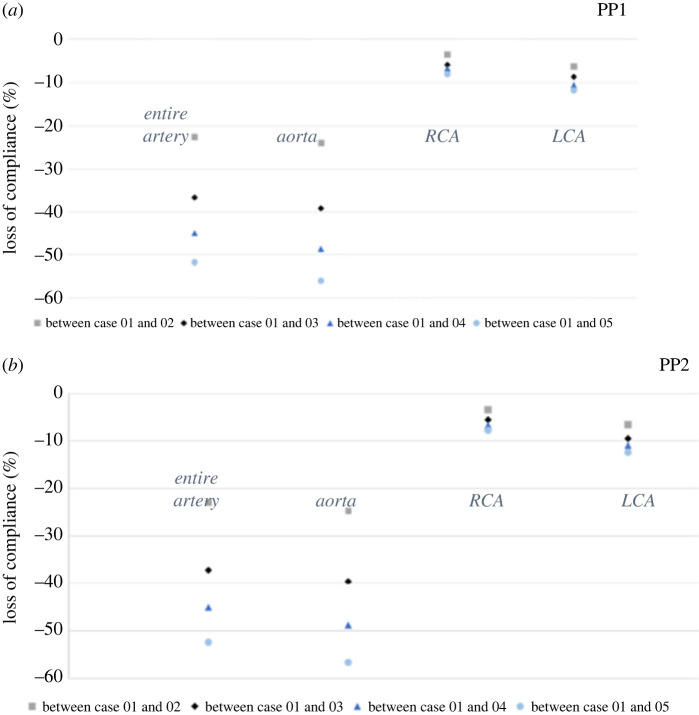
Comparison of change difference (%) in compliance of aorta and carotid arteries in two pulse pressure settings. (*a*) Change difference in compliance at PP of 5994 Pa. (*b*) Change difference in compliance at PP of 7369 Pa. RCA, right carotid artery; LCA, left carotid artery.

Figure 3 shows with a change in pulse pressure a marked loss of compliance between Case 1 and Case 5 of 56% and 52%, respectively. Specifically applied PP (7369 Pa), where the compliance of aorta and aorta and carotid arteries experienced >50% decline from Case 1 to Case 5 (53% and 57% for aorta and aorta and carotid and aorta, respectively).

Above we considered pulse pressure. We next applied variable Young's modulus to the aorta across cases. This enabled us to explore whether fixed aortic Young's modulus can affect either the left or right carotid arteries. We found a similar decrease in the local aortic compliance with an increase in the aortic Young's modulus. In both pressure settings, the left carotid artery was less compliant than the right carotid artery, with a percentage decrease of 12% and 8%, respectively.

Having demonstrated decreased carotid compliance with increasing Young's modulus of the aorta, we further investigated the maximum local compliance changes in both carotid arteries during the normal cardiac cycle. When applying a normal pulse pressure wave of 5994 Pa, we observed a maximum compliance variation between 97 and 99% at the proximal segment of the right common carotid artery, near the bifurcation of the brachiocephalic trunk, as well as the distal segment of the right internal carotid artery. In addition, we observed maximum compliance change for the left carotid artery between 83 and 88%, at the level of the aortic arch bifurcation and the bifurcation of the common carotid artery. Similarly, we observed a maximum compliance change of over 90% with an applied PP of 7369 Pa for both carotid arteries, with the highest compliance change noted at anatomical bends and bifurcations for both carotid arteries.

### 3.3. Regional stress changes for the entire aorta: von Mises stress

**Figure 4.**
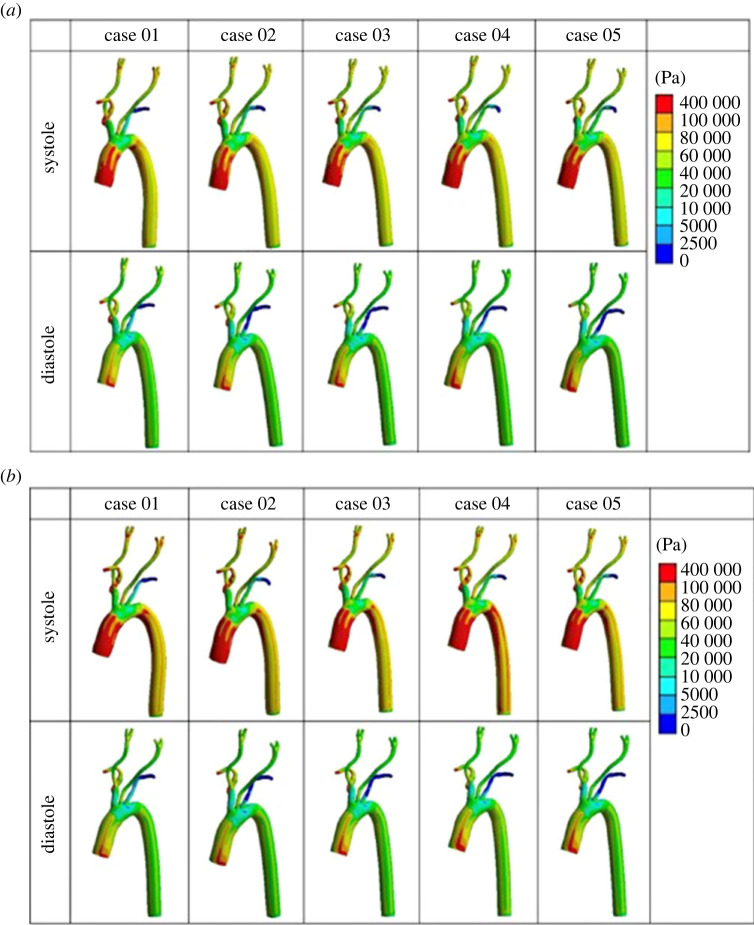
Distribution of von Mises stress on aorta and carotid arteries under two pressure conditions. (*a*) PP = 5594 Pa. (*b*) PP = 7369 Pa.

The three-dimensional distribution of von Mises stress is presented in figure 4.

The von Mises stress distribution on the aortic wall for each case was plotted and observed to provide a visual representation of the computational stresses across our simulated vascular system. The maximum wall stress in both simulation steps increased with the increase of the Young's modulus across the two levels of pulse pressure (electronic supplementary material, figure S3).

### 3.4. Variable stiffness across the aortic arch, ascending and descending aorta

The aortic PWV estimates the aortic stiffness that averages multiple branches of the aortic tree and does not consider the influence of regional differences in the aortic stiffness and diameter. In clinical studies, PWV assessed with MRI method demonstrated different age-related changes of the aorta in the various segments of the aortic tree, including the aortic arch, thoracic and mid descending, and abdominal aorta [23,24]. *In silico* and *in vivo* studies have found that aortic stiffness increased down the aortic tree, with abdominal aorta stiffness mostly affected with increasing age [25,26]. As the aortic stiffness is not uniformly disseminated, in the next step of our simulation model, we aimed to explore the effect of increased aortic stiffness on different segments of the aortic tree, including ascending, descending aorta and the aortic arch.

### 3.5. Regional areas of deformation of the aorta

**Figure 5.**
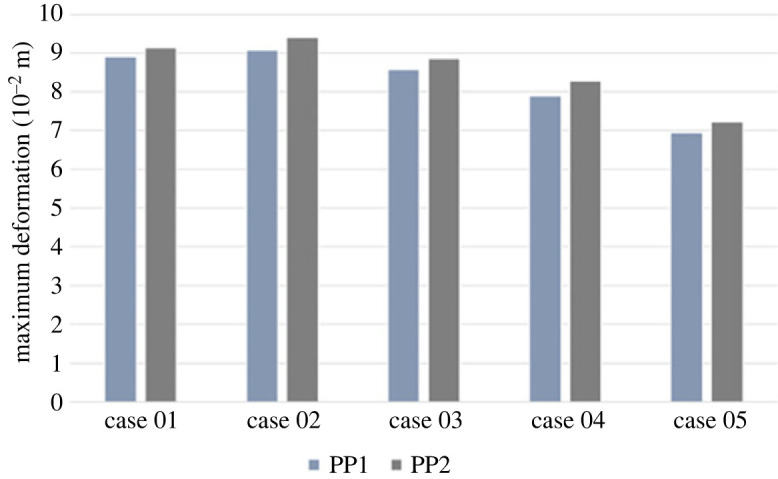
Deformation of aortic segments and carotid arteries with increasing Young's modulus under two pulse pressure conditions: PP1 = 5594 Pa; PP2 = 7369 Pa.

Increasing stiffness for aortic segments is illustrated in figure 5.

Under normal pulse pressure of 5334 Pa being applied, as the Young's modulus increases for the aortic arch, ascending and descending aorta, the maximum deformation decreases, particularly the maximum deformation for Case 1 (9.73 × 10^−2^ (m)) compared to Case 5 (6.71 × 10^−2^ (m)).

Similarly, when increased pulse pressure of 7364 Pa was applied, the maximum deformation of all segments of the aorta decreased with the decrease of the Young's modulus (Case 1 maximum deformation = 9.95 × 10^−2^ (m) compared to Case 5 maximum deformation = 6.89 × 10^−2^ (m)).

### 3.6. Compliance

**Figure 6.**
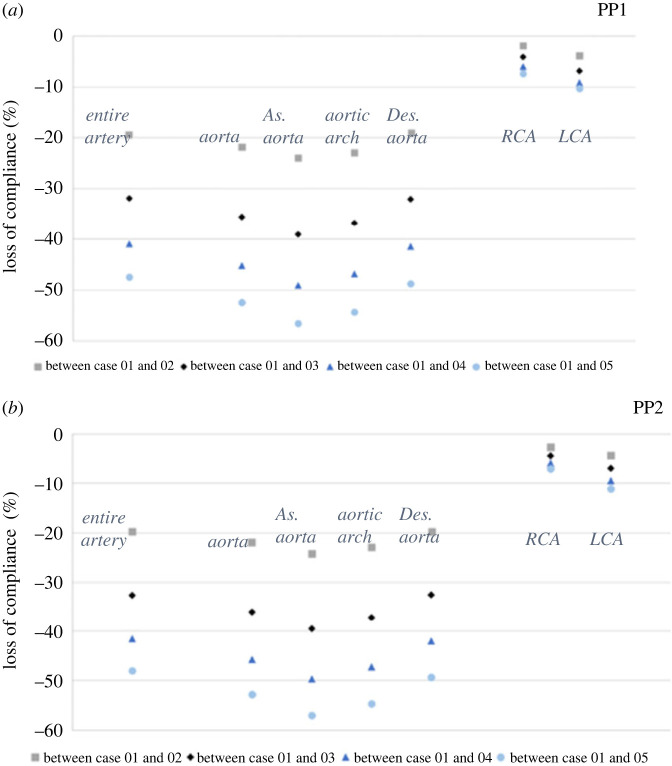
Comparison of change difference (%) in compliance of entire aorta, and carotid arteries in two pulse pressure settings. (*a*) Change difference in compliance at PP of 5994 Pa. (*b*) Change difference in compliance at PP of 7369 Pa.

The compliance of all segments of the aorta and carotid arteries at normal PP (5994 Pa) significantly decreases with the increase of the Young's modulus (electronic supplementary material, figure S5). The highest compliance difference is noted in the ascending aorta and the aortic arch with 57% and 54% decrease between Case 1 and Case 5. When we applied an increased PP (7369 Pa), we noted a maximum decrease in the ascending aorta and aortic arch of 57% and 54%, respectively (figure 6).

After adjusting for both pulse pressure settings as described above, we next applied variable Young's modulus to the different segments of the aorta across cases. This allowed us to examine whether fixed aortic Young's modulus for different regions of the aorta can affect either the left or right carotid arteries. Both left and right carotid arteries showed a decrease in the local compliance with the increase of the aortic Young's modulus. Across both pulse pressures, the left carotid artery was less compliant than the right carotid artery, with a percentage decrease of 11% and 7%, respectively.

As in the first step in our simulation model, we further explored the effect of increasing Young's modulus for the aorta on the maximal local compliance of both carotid arteries. Under the PP of 5994 Pa, the maximum local compliance variation of the right carotid artery was estimated between 96 and 99%, in the middle and distal segments of the common carotid artery and proximal segment of the external carotid artery. For the left carotid artery, we observed maximal local compliance variation between 95 and 99%, with the highest compliance observed in the proximal and distal segments of the common carotid artery. At an applied PP of 7369 Pa, maximum compliance variation of the right carotid artery was between 95 and 99% in the proximal common carotid artery and the distal segments of the external and internal carotid artery. Interestingly, the maximum local compliance for the left carotid artery was 96–99%, presenting at the mid-level of internal and external carotid arteries (data not presented).

### 3.7. Regional stress changes for aortic segments: von Mises stress

**Figure 7.**
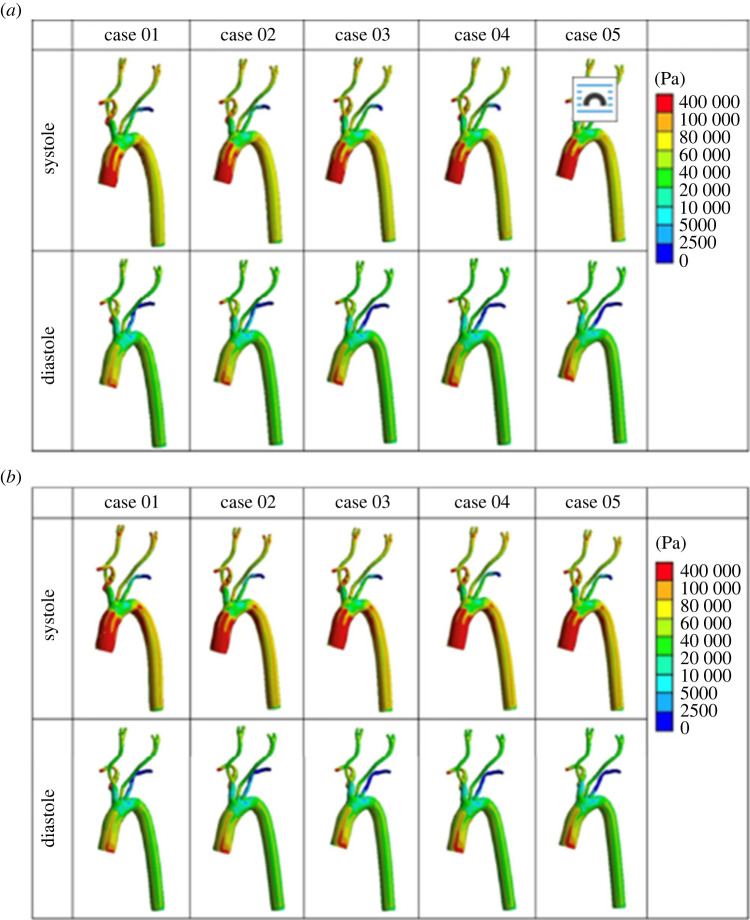
Contour images of the von Mises stress on the arterial wall at systole and diastole under two pulse pressure conditions. (*a*) PP = 5994 Pa. (*b*) PP = 7369 Pa.

The von Mises stress distribution on the aortic wall for each case was plotted and observed to represent and interpret the computational stress analysis results easily (figure 7). The maximum wall stress applied across both PP values increased with the increase of the Young's modulus (electronic supplementary material, figure S6).

## 4. Discussion

Increased stiffness in ageing larger central arteries, such as the aorta and its branches, is associated with cardiovascular risk, including myocardial infarction, heart failure, atrial fibrillation, stroke and renal disease. Arterial stiffness parameters, such as PWV, are also commonly used as predictive values for both all-cause cardiovascular mortality and non-fatal coronary events in diabetic, hypertensive, elderly and community populations [27–29].

While PWV is the gold standard for arterial stiffness measurement, this measure represents an average stiffness value for the entire aortic tree [26,27,30,31]. In addition, there are significant regional differences along the aorta and its branches with respect to variable levels of stiffness, which cannot be fully assessed with the traditional and emerging clinical methods such as ultrasound and applanation tonometry.

Clinical studies have reported variable differences between aortic and carotid stiffness [12,32]. Paini *et al.* reported that the aorta stiffened more than the carotid artery with age [12]. However, the mechanisms for these differences between aortic and carotid stiffness have not been well examined due to a lack of standardized methods and precise mapping of differential effects of aortic stiffness in different segments of the aortic tree.

Our study analysed the complex interactions between different regions of the aortic tree and investigated the influence of aortic stiffness on mechanical behaviours and compliance in carotid arteries.

Specifically, we first evaluated the effect of increasing Young's modulus for the entire aorta on the local compliance and deformation of the carotid arteries. In the first model the aortic stiffness was kept at a constant value at baseline. Secondly, we evaluated the influence of increasing Young's modulus for the entire aorta and subsequently different segments of the aortic tree, including the aortic arch, ascending and descending aorta, while for all models retaining the constant value of carotid Young's modulus at baseline. Finally, we changed the pulse pressure from 5994 Pa to 7639 Pa and explored its effect.

For the variable stiffness across the entire aorta, our data indicate (1) with the increase of Young's modulus, the compliance of the aorta decreases for the aorta (53%) and for both carotid arteries (7% for right and 12% for left carotid artery); (2) maximum local compliance variations between Case 1 and Case 5 were 94–97% for the right and 83–88% for left carotid artery; and (3) compared with baseline, stiffness is decreased when normal and increased pulse pressure is applied.

For the variable stiffness across the aortic tree, our findings highlight the following: (1) with the increase of the Young's modulus for ascending, descending aorta and aortic arch, von Mises stress increased and the deformation and compliance decreased for aorta, all individual segments and both carotid arteries; (2) the maximum local compliance difference varied at 99% for the right and 95–99% for left carotid artery; and (3) again, comparatively stiffness is decreased when normal and increased pulse pressure is applied.

Moreover, we observed decreases in local compliance around the bifurcation and anatomical bends of the carotid arteries when aortic compliance was reduced. Anatomical anomalies such as branching bends and curvatures experience decreased shear stress and turbulent blood flow and have been related as the most vulnerable to the development of atherosclerosis [30,31]. Interestingly, increased stiffness of the carotid wall has been reported in patients with carotid artery dissection, where diagnostic can pose a significant challenge [33–35].

If carotid stiffness increases with accompanying shear stress one would expect an increase in pathological changes over time in these vulnerable areas.

Increased BP, particularly PP, increases pulsative wall stress and is viewed by many authors as an accelerated form of arterial ageing, leading to aortic stiffening [36–38]. However, there is current debate and disagreement regarding the role of arterial stiffness as a predecessor of arterial hypertension. Our results in both simulation steps indicate a similar tendency of increase in aortic and carotid stiffness under normal and increased levels of pulse pressure and support the hypothesis that aortic stiffness precedes the increase of PP. In the Framingham Health Study [39] age related to PP and PWV may not be consistent with the hypothesis that elevated BP is a precursor of aortic stiffening. In this cohort, cfPWV increases from a young age and may be attributable to a concurrent increase in MAP prior to midlife [40] supporting the evidence of a reciprocal relationship between aortic stiffness and hypertension. By contrast, PP, which contributes to the fragmentation of the elastic fibres through a repetitive strain, declines from early adulthood into midlife and then rises again significantly later in life [39]. This pattern of age-related changes suggests that at the individual level, aortic wall stiffening contributes to a substantial increase of PP later in life, which is associated with predominantly systolic hypertension in the elderly [41,42].

To the best of our knowledge, this study is the first to comprehensively analyse the complex relationship between aortic and carotid stiffness and increased aortic stiffness on the mechanical properties and local compliance of the carotid arteries. Cuomo *et al*. conducted a fluid–solid interaction model to explore the effects on ageing, demonstrating an increase in stiffness down the aortic tree in humans aged 40, 60 and 75 years [25]. Xia *et al*. conducted a three-dimensional computational study to explore the aortic stiffness and corresponding haemodynamics on a fixed arterial model. Their results presented increased PWV and cPP but reduced PP amplification, with increased stiffness [43].

Our study has certain limitations, particularly in representing the specific geometry of the aorta and its branches. The anatomy of the aorta, including its bends and bifurcations, can influence stiffness changes along the aortic tree. However, it is important to note that our study was not designed to provide individualized evaluations of aortic and carotid stiffness. In the future, more extensive models derived from diverse clinical cases may help validate our findings across a broader, more diverse populaton. A perceived limitation of our model is that we applied a linear model to the simulations of the aortic and carotid stiffness. While the material model may run into complications for a healthy aorta, we were approximating stiffened vascular circuits. When the aorta becomes stiffer, the elasticity of different layers of the aorta wall may vary in a complex way, depending on individuals, age and measuring approach [44,45]. A fixed linear material property can present a clearer, simple and effective way to get a preliminary idea of the carotid response to changes in aorta stiffness and pressure conditions.

Furthermore, under physiological pressure loading the carotid arteries, nonlinear stiffening with increasing pressure is negligible, and consequently, the linear elastic model better describes the pressure–area dynamics in this vessel [46]. Hence our simulations provide evidence of the effect of aorta stiffness on its downstream arteries quantitatively, with different pulse pressure conditions analysed. However, previous studies have shown that carotid arteries do not exhibit significant pulsatile behaviour compared to the aorta. The choice of a linear elastic material model better predicts its main dynamic properties, as reported in the study by Valdez-Jasso *et al.* [46]. Moreover, our study simulates a model of arteries that are in the range of healthy blood pressure levels (normal low and high pressures); we therefore consider that the arterial wall in our model exhibits limited nonlinear properties.

Although hyperelastic models are often considered to be more accurate predictors of biomechanical characteristics, we noticed that comparative studies between linear elasticity and hyperelasticity models show more or less compatible results, as shown by Kumar *et al*. [47]. The differences between these models are mainly in local wall stress distribution, where hyperelastic models may show higher precision. On the other hand, linear elasticity models offer compelling advantages in terms of simplicity and computational efficiency. And according to Kumar *et al*. [47], the convergence between simulation and experimental results highlights its reliability.

There is a loss of elastic compliance; hence we believe a linear approach better approximates the ageing stiffened aorta with superimposed implications for the at-risk carotid artery, as shown in our simulations. We believe that modelling subtle changes in the elastic healthy aorta may require consideration of a hyperelastic model.

In conclusion, this study presented the first computer simulation of the aorta and carotid arteries to evaluate complex interactions between independent aortic stiffness interactions and carotid mechanical properties. Specifically, the role of deformation and compliance with medical imaging can be used to simulate changes relevant to clinical cases.

The presented simulation models demonstrate a significant interaction of aortic stiffness on the compliance of both carotid arteries. By keeping pulse pressure constant in our models we can provide support for the hypothesis that arterial stiffness may precede the development of systolic hypertension and if BP is controlled with medication aortic stiffness can also interact negatively with distal components of the cerebral vascular tree, including increased vascular resistance and hypoperfusion, leading to ischemia. This study provides future perspectives in computer simulation models for better evaluation and assessment of aortic and local stiffness and application for cardiovascular prevention in clinical practice.

## Data Availability

The data are provided in electronic supplementary material [48].
